# Women’s Groups to Improve Maternal and Child Health Outcomes: Different Evidence Paradigms Toward Impact at Scale

**DOI:** 10.9745/GHSP-D-15-00251

**Published:** 2015-09-10

## Abstract

The Care Group model, with relatively intensive international NGO implementation at moderate scale, appears successful in a wide variety of settings, as assessed by high-quality evaluation with rich program learning. Another women’s group approach—Participatory Women’s Groups—has also been implemented across various settings but at smaller scale and assessed using rigorous RCT methodology under controlled—but less naturalistic—conditions with generally, although not uniformly, positive results. Neither approach, as implemented to date, is directly applicable to large-scale integration into current public programs. Our challenge is to distill the elements of success across these approaches that empower women with knowledge, motivation, and increased self-efficacy—and to apply them in real-world programs at scale.

See related articles by Perry (Care Groups I) and Perry (Care Groups II).

## CARE GROUPS VS. PARTICIPATORY WOMEN'S GROUPS

There is a long history of community-level health education and participatory problem solving in global health. From the late 1990s, two such approaches—Care Groups and Participatory Women’s Groups—have been developed and implemented across a variety of settings and have shown promise.

In this issue of GHSP, we have included two papers documenting program experience to date with Care Groups focusing on maternal and child health.^1^^,^^2^ This model involves use of paid facilitators who, during periodic meetings, deliver focused sets of health messages to Care Group members, who are female community volunteers. These community volunteers, in turn, share the messages with neighboring households.

Similar to Care Groups, Participatory Women’s Groups make use of paid facilitators who meet with female community volunteers (Table). But rather than simply passing on specific health messages, the primary emphasis is on participatory learning and action bearing on factors contributing to poor maternal and newborn outcomes in their community. This model was first piloted in the Warmi project in Bolivia,^3^ which showed reduction in perinatal mortality. Based on this experience, the strategy has subsequently been tested in 7 cluster-randomized controlled trials (RCTs), conducted in Bangladesh, India, Malawi, and Nepal, and has shown promising but mixed results with regard to mortality impact.^4^ Across the 7 trials, approximately 130,000 mother-newborn dyads were enrolled. Based in part on the available RCT evidence from these trials, last year the World Health Organization (WHO) formally endorsed this approach.^5^

**TABLE:** Comparing Care Groups With Participatory Women’s Groups

	Care Groups	Participatory Women’s Groups
**Skilled external facilitators**	Required	Required
**Importance of group interaction**	Contributes to effectiveness	Central focus
**Health message communication**	Central focus	Secondary focus
**Role of women’s group members**	Peer health educators to neighboring households	Problem solvers developing community solutions
**Population coverage strategy**	Blanket coverage, through group members, of households with pregnant women and children under 5	Initially no explicit strategy aiming at coverage, but—based on RCT results—for achieving impact, recommend reaching ≥30% of pregnant women

Experience with Care Groups is not captured as well in the peer-reviewed literature, but—as with the Participatory Women’s Group approach just described—these programs also trace their origin to the late 1990s. The model was first developed and tested by World Relief in Mozambique, under the United States Agency for International Development (USAID) Child Survival and Health Grants Program.^6^ The approach has been picked up by at least 24 other international NGOs and has been implemented in 28 countries.^1^ Across a selection of 8 of these programs reported in the paper by Perry et al.,^1^ the total number of beneficiaries was 738,000. Overall, experience with such programs has consistently suggested mortality impact, albeit with less rigorous study designs.

## DIFFERENT KINDS OF EVIDENCE

Although the two approaches were introduced around the same time and are similar in some respects, the course they’ve charted over the past decade and a half has differed markedly. With the peer-reviewed publication of 7 cluster RCTs on Participatory Women’s Groups, this approach has now accrued an “evidence base” and, as mentioned, has given rise to WHO guidance. By contrast, the Care Group model has accumulated what could be described as a reasonably large and fairly well-documented (although not well-published) “experience base.”

The inputs applied have varied across settings but, for both of these initiatives, have been reasonably significant, exceeding what the public sector could or would otherwise have provided to the populations targeted for such activities. Although some of the Participatory Women’s Group trials enrolled up to 30,000 mother-newborn dyads, overall the reach of program efforts using the Care Group model has exceeded that of Participatory Women’s Groups by at least *an order of magnitude*.

The Participatory Women’s Group program experiences, however, have been evaluated using rigorous population vital registration and RCT designs, allowing for firmer inferences on mortality effects. So with regard to “internal validity,” the available evidence allows us more confidently to conclude that, “It worked *there*,”^7^ at least in the particular settings of the trials that showed a mortality effect. By contrast, the Care Group program experiences do not have the benefit of systematic mortality documentation, and—although they used pre-post designs with important service utilization and household practice endpoints in all the documented instances—generally they did *not* use designs tracking pre-post changes in an equivalent comparison area. As such, they do not meet the conventional methodologic threshold for “high-quality evidence” generally used by global normative bodies such as WHO for developing program guidance (which, in its evidence reviews, often discounts studies lacking concurrent comparison arms).

## “RIGOR” VS. RELEVANCE

What Care Group projects lacked in conventional “rigor,” they have made up for as more “naturalistic” program experiences and as such are, arguably, *more broadly relevant* for decision making in the real world. In most instances, the Care Group projects were implemented district-wide, usually linking with government health services. The intensity of inputs per beneficiary reached was generally significantly more modest than in the Participatory Women’s Group RCTs. Of course, for the documented Care Group experiences, with the lesser degree of rigor in measurement and design, we need to approach causal claims more tentatively. But the consistency in improved household practices and service utilization across cases strongly suggests that, providing that conditions for effective implementation are met, the Care Group approach yields mortality reduction benefit.

What Care Group evaluations lacked in “rigor,” they have made up with “naturalistic” program experiences, making the findings arguably more relevant for decision making.

The authors of the two Care Group papers in this issue suggest that a more rigorous RCT design may be needed to test the Care Group approach. *We disagree.* Under current institutional norms, there may not be WHO recommendations forthcoming for a Care Group approach without evidence derived from stronger research methodology than what has been used to date (although the WHO guidelines development process does not automatically exclude non-experimental studies). Nevertheless, funders and other decision makers already have a sufficiently solid “experience base,” if not an RCT-grounded “evidence base,” for the Care Group approach to warrant further such program work.

## THE RIGHT PARADIGM FOR SCALING-UP COMPLEX INTERVENTIONS?

The current tension between contesting views of what constitutes a valid source of insight to inform decision making suggests that some are getting hung up on a misapplied paradigm. The RCT methodology is very useful in isolating the effect of a particular intervention from other possible contributing effects. But RCTs often trade off real-world generalizability (and utility) for internal validity, *particularly when not complemented with other information*.^8^ Even if an RCT gives us considerable confidence that “it worked *there*” (often under very case-specific conditions), it doesn’t necessarily tell us much about whether it would work *here*, i.e., in the range of other settings where policy makers and funders may be considering a similar effort.^7^ In particular, that a program approach worked under very controlled and rigorously implemented conditions tells us little about how it would perform under usual ministry of health program conditions.

Even if RCTs give us confidence that “it worked *there*,” they don’t necessarily tell us much about whether it would work *here*.

The dominant evidence paradigm in global health, as illustrated in the Figure, starts with the question, “Does it work?” It seeks a universal, context-free answer, asking, “Is it effective, yes/no?” According to this paradigm, the definitive answer to this question is obtained from an RCT or, even better, from a multi-center trial or a systematic review of multiple RCTs.^9^ On the basis of a positive answer to this “efficacy” question, one proceeds directly to widespread adoption. For a discrete clinical intervention (e.g., innovative cancer regimen vs. standard regimen), this paradigm may be appropriate. However, generally it is inadequate for behavioral interventions or complex, multi-element approaches or strategies, for which contextual factors are usually very important.^8^

**FIGURE:**
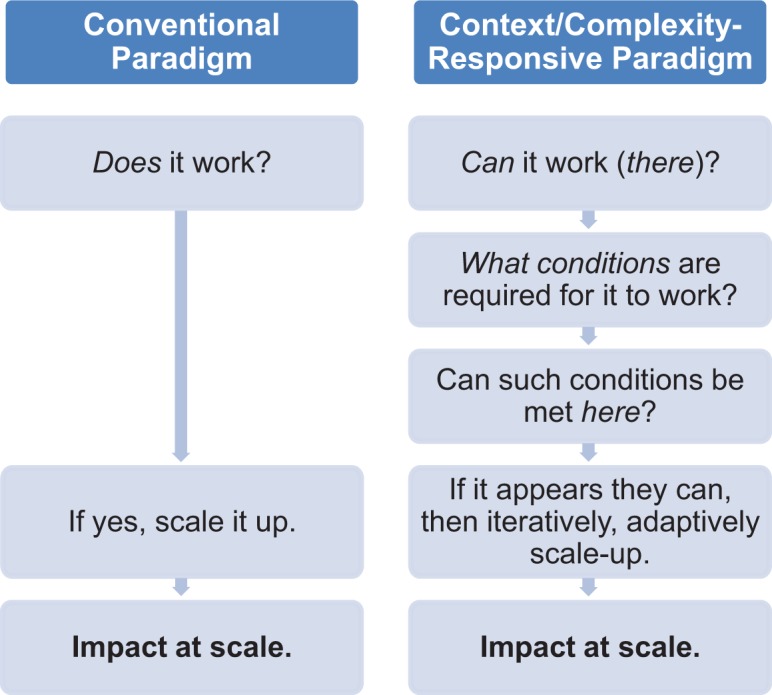
An Appropriate Paradigm for “Evidence” and Scale-Up of Complex Interventions

Instead, more appropriate for behavioral interventions and multi-element intervention packages—that are overwhelmingly the focus of global health program work—we need a paradigm that is conscious of and responsive to context and complexity. Instead of accepting the notion that all we need is “evidence” of effectiveness (understood as universal and context-free), such a paradigm recognizes both the utility and limitations of RCT evidence as generally demonstrating only that an intervention or approach *can* work in the particular conditions and setting under which the original trial(s) was or were conducted.^7^ Beyond such proof of concept, to make judgments on whether the intervention or strategy could subsequently work in the program setting where it is being considered, we then need to understand, beyond the intervention itself, what the needed support factors were to produce the effect observed in the original study or studies (what Cartwright refers to as “lateral search”^7^).

## HOW BEST TO APPLY EVIDENCE?

*For both Participatory Women’s Groups and Care Groups, we now have reason to believe they can be effective, but also that neither of these approaches is necessarily or automatically effective*. So, it is inappropriate simply to label *either* of these as “evidence-based” and then enjoin governments and donors to “get with the program” and roll them out (as our usual paradigm prescribes). Instead, for both we need to better understand, based on evidence available and experience to date, what set of conditions needs to be met for effectiveness. Then, in each particular setting where government, donors, or others are considering similar program efforts, there needs to be a process of exploring what it might take to meet the needed conditions, as best as we understand them. Early tentative steps can then be taken, validating to what degree we’re actually achieving what we had hoped to for optimal performance. In most instances, we’ll find gaps between expected and actual performance; *no battle plan survives contact with the enemy*. In some cases, we may determine that in our particular setting, in the way we’ve tried to implement a particular solution, it won’t give us what we want. In such cases, the best choice may be *to cut our losses and try something else*. In other cases, certain aspects may perform well and others less well—and the best choice may be to adapt the approach.

For both Participatory Women’s Groups and Care Groups, we have promising indications that—under the right conditions—important shifts in behavior can be achieved, resulting in population-level improvements in maternal, newborn, or child health outcomes, as well as other benefits. In both instances, tentative efforts in new settings and through new implementation modalities (possibly including under government primary health care services) are warranted—but not under the paradigm of “efficacy established; scale it up.”

“Positive RCT result → efficacy established → scale it up.” Maybe we’re missing a step or two.

Unfortunately neither the Participatory Women’s Group nor the Care Group approach is a natural fit for typical primary health care services in low- and middle-income countries. However, in both cases there may be key principles or practices that can be abstracted and applied in programs in ways that may look quite different from the original two models but that may fit the conditions in a new receiving system and deliver benefit. Is there some “secret sauce” for empowering women with knowledge, motivation, and increased self-efficacy that we can draw out of these experiences that might be applied in some more practicable way in current programming?

## WHERE NEXT?

Beyond RCTs, there is a need for well-documented and well-evaluated “incubator” experiences, experiences intermediate between proof-of-concept cluster RCTs (as conducted for Participatory Women’s Groups) and large-scale implementation under ministries of health. The Care Group experience documented in the two papers in this issue provides a good example of such an incubator experience. At this point, one can say that through NGO delivery modalities and with significant external resources, the Care Group approach can be effective across a fairly wide range of settings. Moving to a further phase of (well-studied) incubation, efforts could now be made to tap the power of women’s groups to improve maternal and child health through larger-scale Care Group efforts under ministries of health.

To inform global health programming, we need “incubator” experiences—intermediate experiences between proof-of-concept RCTs and large-scale implementation under ministries of health.

Of course, there are many worthwhile initiatives that continue to produce widespread benefit without being delivered through government health services. Regardless of whether either of these approaches is ever demonstrated to perform effectively in such an institutional context, both demonstrate a potentially powerful local dynamic that could be tapped in many settings—women meeting and taking action together for a better future for themselves, their families, and their communities. *–Global Health: Science and Practice*

## References

[1] Perry H Morrow M Borger S Weiss J DeCoster M Davis T Care Groups I: an innovative community-based strategy for improving maternal, neonatal, and child health in resource-constrained settings. Glob Health Sci Pract. 2015;3(3):358–369. 10.9745/GHSP-D-15-00051 PMC4570011 26374798

[2] Perry H Morrow M Davis T Borger S Weiss J DeCoster M Care Groups II: a summary of the child survival outcomes achieved in high-mortality, resource-constrained settings using volunteer health workers. Glob Health Sci Pract. 2015;3(3):370–381. 10.9745/GHSP-D-15-00052 PMC4570012 26374799

[3] O'Rourke K Howard-Grabman L Seoane G. Impact of community organization of women on perinatal outcomes in rural Bolivia. Rev Panam Salud Publica. 1998;3(1):9–14. 9503957 10.1590/s1020-49891998000100002

[4] Prost A Colbourn T Seward N Azad K Coomarasamy A Copas A Women's groups practising participatory learning and action to improve maternal and newborn health in low-resource settings: a systematic review and meta-analysis. Lancet. 2013;381(9879):1736–1746. 10.1016/S0140-6736(13)60685-6. 23683640 PMC3797417

[5] World Health Organization (WHO) WHO recommendation on community mobilization through facilitated participatory learning and action cycles with women’s groups for maternal and newborn health. Geneva: WHO; 2014 Available from: http://www.who.int/maternal_child_adolescent/documents/community-mobilization-maternal-newborn/en/25165805

[6] Edward A Ernst P Taylor C Becker S Mazive E Perry H. Examining the evidence of under-five mortality reduction in a community-based programme in Gaza, Mozambique. Trans R Soc Trop Med Hyg. 2007;101(8):814–822. 10.1016/j.trstmh.2007.02.025. 17482222

[7] Cartwright N Hardie J Evidence-based policy: a practical guide to doing it better. New York: Oxford University Press; 2012.

[8] Shelton JD. Evidence-based public health: not only whether it works, but how it can be made to work practicably at scale. Glob Health Sci Pract. 2014;2(3):253–258. 10.9745/GHSP-D-14-00066. 25276583 PMC4168632

[9] Pawson R Systematic obfuscation: a critical analysis of the meta-analytic approach. In: Evidence-based policy: a realist perspective. Thousand Oaks (CA): SAGE Publications; 2006.

